# The New Building of the Dental Department of the University of Maryland

**Published:** 1884-07

**Authors:** 


THE NEW BUILDING OF THE DENTAL DEPARTMENT OF THE UNIVERSITY OF MARYLAND, is now about completed, and presents a structure imposing in appearance and arrangements, having been erected especially for instruction in practical dentistry. Another great advantage is that this fine building is owned by the University, and is erected on its grounds, in such a location as commands unobstructed light from *every* side. Notwithstanding the obstruction incident to the erection of new structures, the Infirmary practice has been exceedingly large throughout the entire spring and summer, and the class in attendance has had all the practical work both in operative and mechanical dentistry that could possibly be attended to. The dental students have also the advantage of attending the daily clinics at the University Hospital on Oral and General Surgery, etc., etc., throughout the spring and summer.

